# Battle of the Biofuels

**DOI:** 10.1289/ehp.115-a92

**Published:** 2007-02

**Authors:** John Manuel

With skyrocketing petroleum prices and war in the oil-producing nations of the Middle East, biofuels are increasingly touted as desirable alternatives to petroleum. But can they really help free us from a petroleum economy? How do they compete with conventional fuels and each other on a cost basis? What are the environmental impacts? Researchers at the University of Minnesota have published a wide-ranging study that offers some answers.

Currently, corn grain ethanol and soybean biodiesel are the two predominant alternative transportation fuels in the United States. Both can be used in conventional car and truck engines in blended form, and biodiesel can also be used in pure form (“B100”). Both are available at an increasing number of wholesale and retail locations across the nation. However, both require significant energy to produce, have their own environmental impacts, and could divert corn and soybeans from the nation’s food supply. Exactly what the energy balance and environmental impacts are and whether these fuels should be subsidized has been the subject of heated debate among scientists, policy makers, and the public.

Researchers from the University of Minnesota and St. Olaf College led by ecology professor G. David Tilman hoped to inform this debate by conducting a comprehensive analysis of the full life cycles of these biofuels. According to the study, published in the 25 July 2006 issue of the *Proceedings of the National Academy of Sciences*, a viable alternative fuel must meet four criteria: show superior environmental benefits over the fossil fuel it displaces, be economically competitive with that fossil fuel, be producible in sufficient quantities to make a meaningful impact on energy demands, and provide a net energy gain over the energy sources used to produce it.

## Comparison: The Study Findings

The authors performed their analysis using public data on farm yields, commodity and fuel prices, farm energy and chemical inputs, production plant efficiencies, production of coproducts (e.g., an animal feed known as distillers dried grains with solubles, or DDGS), greenhouse gas emissions, and other environmental effects. The boundaries the authors established in accounting for energy inputs were larger than in other studies, including, for example, the energy required to manufacture the machinery used to farm corn and soybeans.

The analysis showed that both corn grain ethanol and soybean diesel have a positive net energy balance (NEB), with ethanol having a 25% NEB and biodiesel a 93% NEB. That is, ethanol yields 25% more energy than is required to produce it, and biodiesel yields 93% more. At the same time, the analysis showed that ethanol’s positive NEB is attributable largely to the energy credit for DDGS rather than to the ethanol itself. Coauthor Jason Hill explains, “In our paper, the energy credit [for DDGS] is offered primarily because this product allows for offsetting the production of other products—such as corn and soybean meal—as it can replace a combination of these in the market.”

“Corn grain ethanol has a low NEB because of the high energy input required to produce corn and to convert it into ethanol,” the authors wrote. Soybean diesel, on the other hand, requires far less energy to convert biomass to biofuel “because soybeans create long-chain triglycerides that are easily expressed from the seed.”

The study showed that the cultivation of both corn and soybeans for biofuels creates negative environmental impacts, most notably the leaching of pesticides as well as nitrogen and phosphorus from fertilizers, into surface, ground, and coastal waters. Corn production involves markedly greater releases of nitrogen, phosphorus, and pesticides than does soybean production. Moreover, the pesticides used in corn production tend to be more environmentally harmful and persistent than those used to grow soybeans.

Ethanol also fares worse than biodiesel when it comes to the emission of air pollutants and greenhouse gases compared with their petroleum counterparts. The study found that biodiesel reduces carbon dioxide emissions by 41% compared with conventional diesel, whereas ethanol yields only a 12% reduction compared with gasoline.

The use of ethanol as an oxygenate has long been touted as reducing air pollution, and the study did find that 10% ethanol used as an additive instead of methyl *tert*-butyl ether (MTBE) can reduce emissions of carbon monoxide, volatile organic compounds, and particulate matter with a diameter of less than 10 μm. However, because of the emissions involved in the production, transportation, and conversion of corn into ethanol, the popular E85 ethanol–gasoline blend (which is 85% ethanol and 15% gasoline) results in higher total life-cycle emissions of these three pollutants as well as of oxides of sulfur and nitrogen.

Conversely, biodiesel blended into diesel at low levels reduces emissions of volatile organic compounds, carbon monoxide, particulate matter, and sulfur oxides during combustion. Further, over the full life cycle, biodiesel blends reduce carbon monoxide, particulate matter, and sulfur oxides compared with diesel.

The authors stated that in 2005, when the study was conducted, neither biofuel was cost-competitive with petroleum-based fuels without subsidy. (The federal government provides subsidies of 20¢ per energy equivalent liter [EEL] for ethanol and 29¢ per EEL for biodiesel. Various states provide additional subsidies.) According to the article, corn grain ethanol cost 46¢ per EEL to produce, while gasoline averaged 44¢ per liter to produce. Soybean diesel cost 55¢ per EEL to produce, while diesel production prices averaged 46¢ per liter. According to coauthor Erik Nelson, production costs have risen since the paper’s publication to about 63¢ per EEL for ethanol and 82¢ per EEL for biodiesel versus 45¢ per liter for gasoline and 53¢ per liter for diesel.

The study also examined the potential of corn and soybeans to meet U.S. demand for transportation fuel. It found that devoting all U.S. corn and soybean production to ethanol and biodiesel would have offset only 12% and 6% of U.S. gasoline and diesel demand, respectively, in 2005. And because of the increased use of fossil fuels needed to produce this amount of biofuel, net energy gain would be reduced to just 2.4% for ethanol and 2.9% for biodiesel.

The authors concluded that soybean biodiesel has major advantages over corn grain ethanol, but that neither can be depended upon to significantly reduce our reliance on petroleum without competing with the food supply. Instead, the report called for the development of biofuels based on nonfood crops such as prairie grasses and woody plants, which can be converted to synthetic hydrocarbons or cellulosic ethanol.

## Response from the Field

Peter Ciborowski, a research scientist with the Minnesota Pollution Control Agency, notes that although there have been several studies on the costs and benefits of ethanol, the Minnesota study is important for its new information on the environmental impacts of biodiesel. Ciborowski also notes the significance of the finding that complete conversion of the U.S. corn crop to ethanol would meet only 12% of U.S. transportation fuel needs and yield only a 2.4% net energy gain. “This has obvious implications for a national biofuel-based response to the energy security problem using corn-based ethanol as the basis for the strategy: the numbers simply don’t work,” he says. “That suggests that the most sensible national use of what will always be a limited supply might be as an oxygenate in place of MTBE or in mixtures of [10–20% ethanol blended with 80–90% gasoline].”

Other reactions have been less positive. The National Corn Growers Association is critical of the study’s assessment of the NEB of corn grain ethanol. Citing a 2004 USDA report titled *The 2001 Net Energy Balance of Corn-Ethanol*, the association claims that corn grain ethanol has an NEB of 67% rather than 25%. Hill responds that his team employed information from the USDA report in their assessment, but added data on other inputs to provide what they believed to be a more accurate accounting.

The group also criticizes the assumption that increasing the production of corn to make ethanol will have negative effects on the environment. “Data very clearly supports our statement that chemical usage has not increased in any significant fashion even though production [of corn] has increased,” says Geoff Cooper, director of commercialization and business development with the National Corn Growers Association. “That’s because the introduction of new insect- and weed-resistant traits of corn are decreasing the need for chemical inputs.”

David Morris, vice president of the nonprofit Institute for Local Self-Reliance, which promotes local generation and ownership of energy sources, does not dispute the report’s major findings but questions their significance. “Its primary findings are neither new nor controversial,” Morris says. “The net energy of soybean-derived diesel is much better than corn-derived ethanol, and cellulosic material promises to be better than both.”

All parties consulted agree that the future for biofuels lies in the ability to convert largely nonfood-based cellulosic materials to fuel. In a follow-up study published in the 8 December 2006 issue of *Science*, Tilman, Hill, and fellow University of Minnesota researcher Clarence Lehman continued the search for such a crop, identifying low-input high-diversity mixtures of native grasses that provide more usable energy, fewer greenhouse gas emissions, and less agrichemical pollution per hectare than either corn grain ethanol or soybean biodiesel. This study contends that these low-input high-diversity biofuels can be produced on agriculturally degraded lands and thus will not displace food production or cause loss of biodiversity via habitat destruction.

“Producing biofuel for transportation is a fledgling industry,” says Tilman. “Corn ethanol and soybean diesel are successful first-generation biofuels. The next step is a biofuel crop that requires low chemical and energy inputs and can give us greater energy and environmental returns.”

## Figures and Tables

**Figure:**
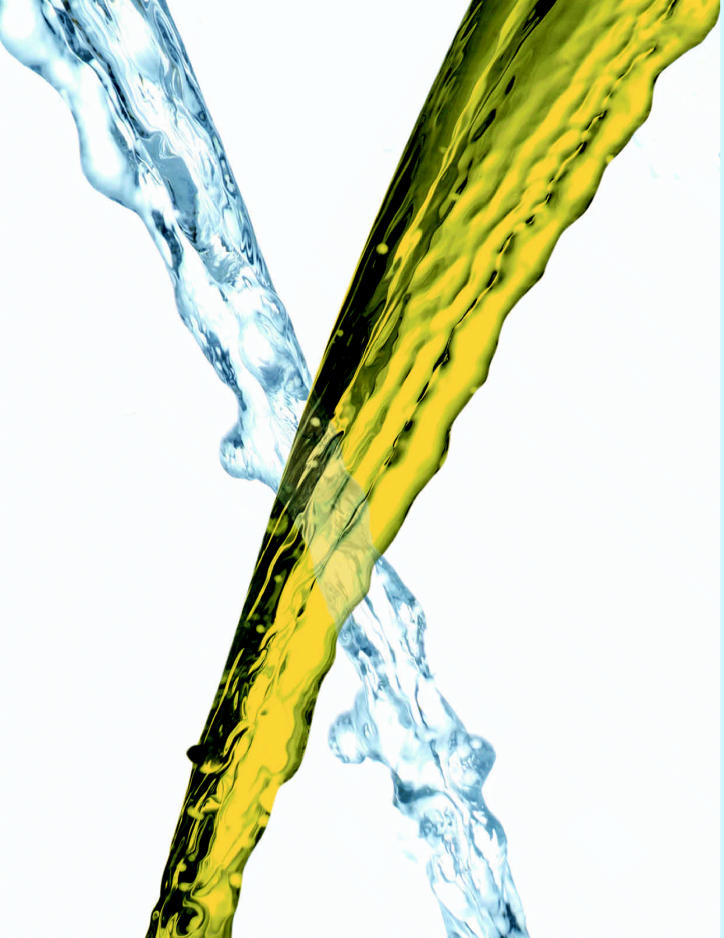


**Figure:**
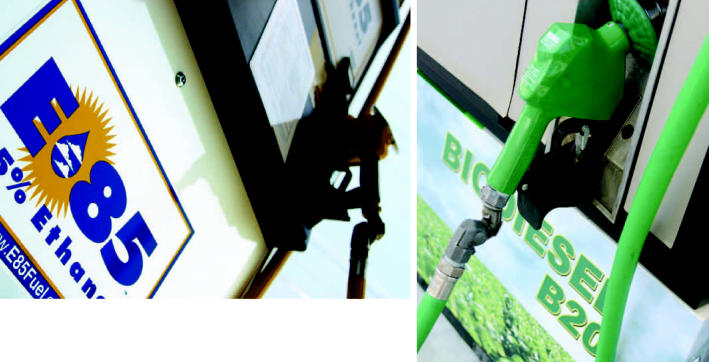
No clear winner In a comparison of the environmental and economic benefits of ethanol and biodiesel, the latter has a slight edge. However, neither alternative provides a clear solution to the nation’s energy problem.

